# Crystal structure of bromido­nitro­syl­bis(tri­phenyl­phosphane-κ*P*)nickel(II)

**DOI:** 10.1107/S2056989015004703

**Published:** 2015-03-18

**Authors:** Rose Hockley, Hira Irshad, Tippu S. Sheriff, Majid Motevalli, Sarantos Marinakis

**Affiliations:** a Department of Chemistry and Biochemistry, School of Biological and Chemical Sciences, Queen Mary University of London, Joseph Priestley Building, Mile End Road, London E1 4NS, England

**Keywords:** crystal structure, nickel complex, nitrosyl complex, tri­phenyl­phosphane ligand, hydrogen bonds, C—H⋯π inter­actions

## Abstract

The asymmetric unit of the title complex, [NiBr(NO){P(C_6_H_5_)_3_}_2_], comprises two independent mol­ecules each with a similar configuration. The Ni^II^ cation is coordinated by one bromide anion, one nitrosyl anion and two tri­phenyl­phosphane mol­ecules in a distorted BrNP_2_ tetra­hedral coordination geometry. The coordination of the nitrosyl group is non-linear, the Ni—N—O angles being 150.2 (5) and 151.2 (5)° in the two independent mol­ecules. In the crystal, mol­ecules are linked by weak C—H⋯Br hydrogen bonds and weak C—H⋯π inter­actions into a three-dimensional supra­molecular architecture.

## Related literature   

For general background to transition metal nitro­syls, see: Westcott & Enemark (1999 ▸); De La Cruz & Sheppard (2011 ▸). For the structures of closely related compounds, see: Enemark (1971 ▸); Haller & Enemark (1978 ▸). For the synthesis of the title complex, see: Feltham (1960 ▸, 1964 ▸).
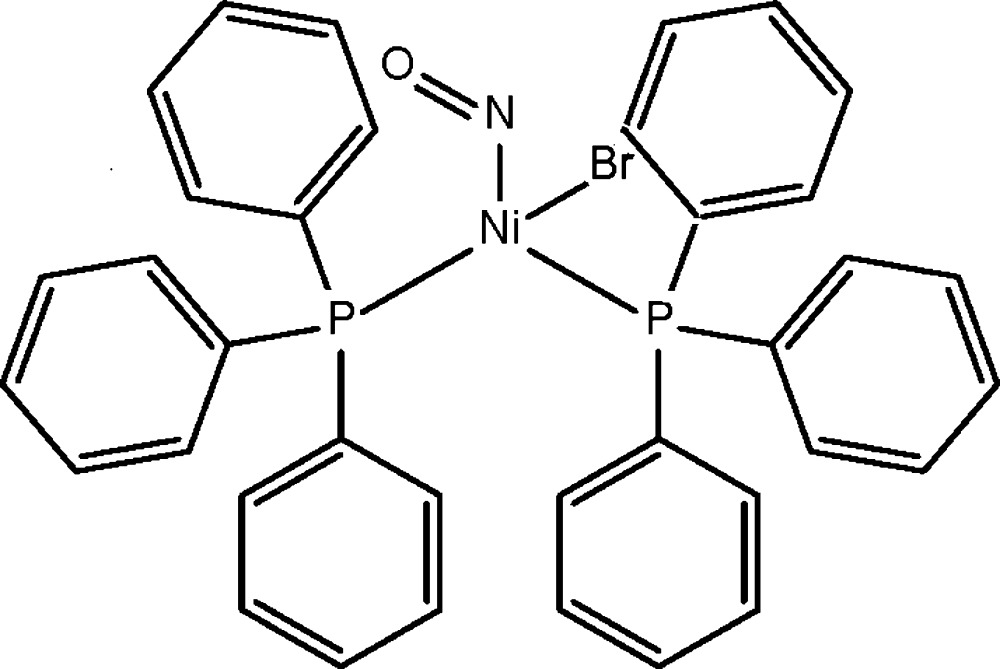


## Experimental   

### Crystal data  

[NiBr(NO)(C_18_H_15_P)_2_]
*M* *_r_* = 693.17
Triclinic, 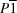

*a* = 9.5585 (6) Å
*b* = 14.7675 (7) Å
*c* = 22.6079 (12) Å
α = 79.708 (4)°
β = 85.771 (4)°
γ = 89.944 (4)°
*V* = 3131.1 (3) Å^3^
*Z* = 4
Mo *K*α radiation
μ = 2.03 mm^−1^
*T* = 100 K
0.2 × 0.1 × 0.04 mm

### Data collection  

Rigaku HG Saturn724+ diffractometer
Absorption correction: multi-scan (*CrysAlis PRO*; Agilent, 2014 ▸) *T*_min_ = 0.82, *T*_max_ = 0.92
29886 measured reflections
17527 independent reflections
14543 reflections with *I* > 2σ(*I*)
*R*_int_ = 0.131

### Refinement  

*R*[*F* ^2^ > 2σ(*F* ^2^)] = 0.050
*wR*(*F* ^2^) = 0.125
*S* = 1.04
17491 reflections
758 parameters
H-atom parameters constrained
Δρ_max_ = 0.77 e Å^−3^
Δρ_min_ = −0.61 e Å^−3^

### 

Data collection: *CrysAlis PRO* (Agilent, 2014 ▸); cell refinement: *CrysAlis PRO*; data reduction: *CrysAlis PRO*; program(s) used to solve structure: *SHELXS2014* (Sheldrick, 2008 ▸); program(s) used to refine structure: *SHELXL2014* (Sheldrick, 2015 ▸); molecular graphics: *ORTEP-3 for Windows* (Farrugia, 2012 ▸); software used to prepare material for publication: *PLATON* (Spek, 2009 ▸).

## Supplementary Material

Crystal structure: contains datablock(s) I, global. DOI: 10.1107/S2056989015004703/xu5839sup1.cif

Structure factors: contains datablock(s) I. DOI: 10.1107/S2056989015004703/xu5839Isup2.hkl

Supporting information file. DOI: 10.1107/S2056989015004703/xu5839Isup3.mol

Supporting information file. DOI: 10.1107/S2056989015004703/xu5839Isup4.cdx

**Fig. 1.**
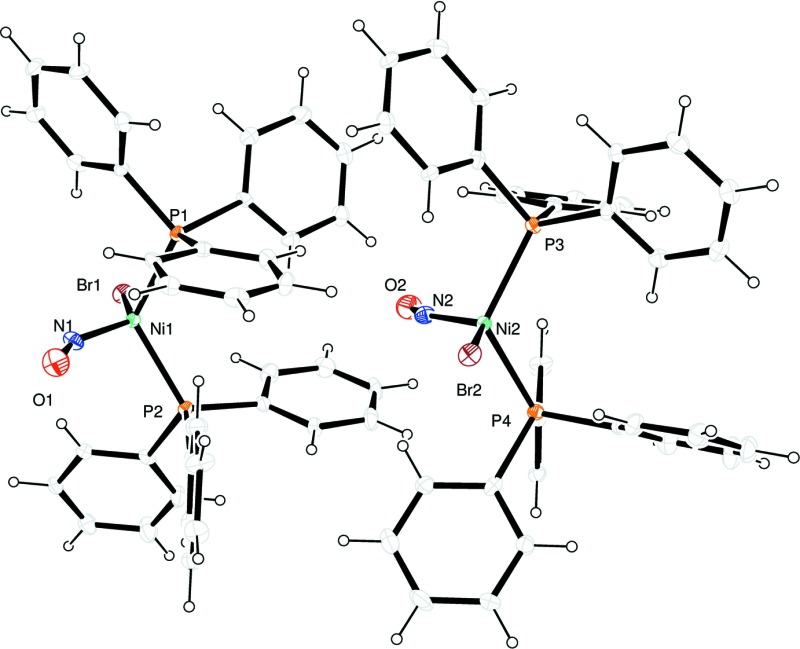
The molecular structure of the title complex, with atom labels and 50% probability displacement ellipsoids for non-H atoms.

. DOI: 10.1107/S2056989015004703/xu5839fig1.tif
The mol­ecular structure of the title complex, with atom labels and 50% probability displacement ellipsoids for non-H atoms.

**Fig. 2.**
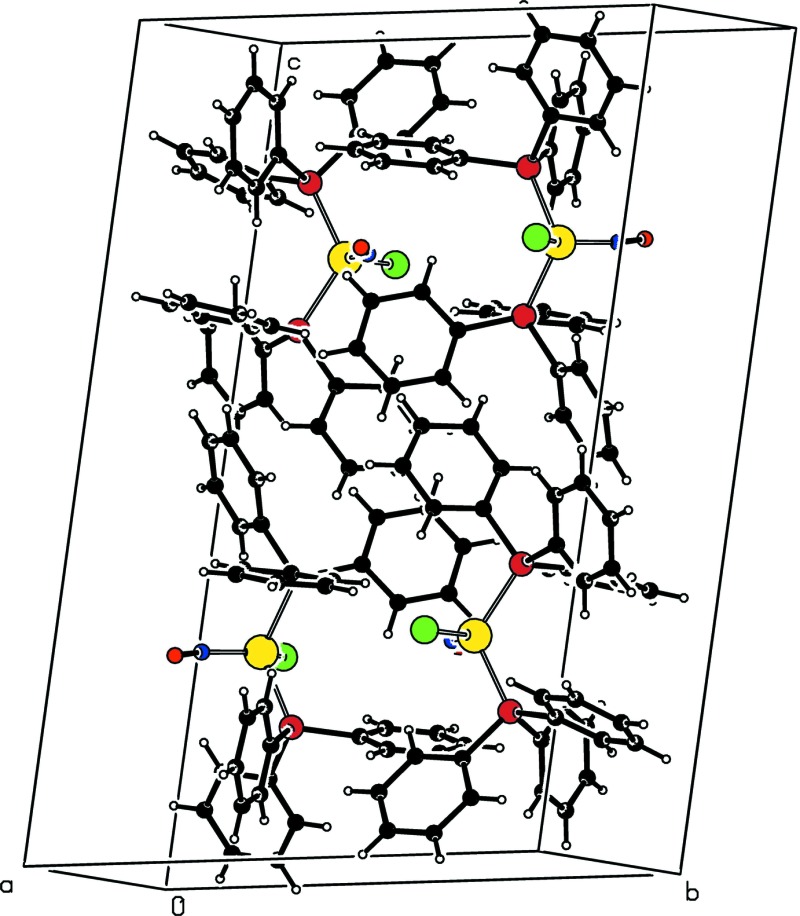
The packing of the title complex.

. DOI: 10.1107/S2056989015004703/xu5839fig2.tif
The packing of the title complex.

CCDC reference: 1052684

Additional supporting information:  crystallographic information; 3D view; checkCIF report

## Figures and Tables

**Table 1:** Hydrogen-bond geometry (, ) *Cg*2, *Cg*4, *Cg*6 and *Cg*8 are the centroids of the C7C12, C19C24, C31C36 and C43C8 rings, respectively.

*D*H*A*	*D*H	H*A*	*D* *A*	*D*H*A*
C2H2Br1	0.95	2.90	3.779(7)	155
C9H9Br1^i^	0.95	2.85	3.588(6)	135
C38H38Br2	0.95	2.88	3.777(7)	157
C45H45Br2^ii^	0.95	2.89	3.622(6)	135
C72H72Br2	0.95	2.85	3.706(6)	150
C18H18*Cg*4	0.95	2.88	3.681(7)	143
C30H30*Cg*2	0.95	2.65	3.487(7)	147
C35H35*Cg*4^iii^	0.95	2.90	3.711(7)	145
C56H56*Cg*8	0.95	2.67	3.523(7)	149
C64H64*Cg*6^iv^	0.95	2.69	3.490(7)	142

Symmetry codes: (i) 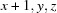; (ii) 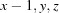; (iii) 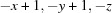; (iv) 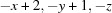.

## supplementary crystallographic information

### S1. Chemical context

For general background to transition metal nitro­syls, see: Westcott & Enemark (1999); De La Cruz & Sheppard (2011). For the structure of closely related compounds, see: Enemark (1978); Haller & Enemark (1978). For the synthesis of the title complex, see: Feltham (1960); Feltham (1964).

### S2. Structural commentary

In the title molecular complex, [NiBr(NO)(P(C_6_H_5_)_3_)_2_], the crystal structure consists of two discrete molecules with distorted tetra­hedral coordination geometry about the Ni atom. The coordination of the nitro­syl group is distinctly non-linear with an averaged value of 150.7 (5)° for the Ni—N—O angle. The (average) value for the P—Ni—P angle is 121.63 (6)°, and the Br—Ni—N angle is 126.40 (17)°. Important inter­atomic (average) distances are Ni—P 2.2423 (17) and 2.2584 (17) Å, Ni—Br = 2.3979 (9) Å, Ni—NO = 1.692 (5) Å, and N—O = 1.152 (6) Å. Although this compound has been previously synthesized (Feltham, 1960), details of the crystal structure have not been presented.

### Crystal data

**Table:**

[NiBr(NO)(C_18_H_15_P)_2_]	*Z* = 4
*M**_r_* = 693.17	*F*(000) = 1416
Triclinic, *P*1	*D*_x_ = 1.470 Mg m^−^^3^
Hall symbol: -P 1	Mo *K*α radiation, λ = 0.71073 Å
*a* = 9.5585 (6) Å	Cell parameters from 10711 reflections
*b* = 14.7675 (7) Å	θ = 2.2–27.4°
*c* = 22.6079 (12) Å	µ = 2.03 mm^−^^1^
α = 79.708 (4)°	*T* = 100 K
β = 85.771 (4)°	Plate, black
γ = 89.944 (4)°	0.2 × 0.1 × 0.04 mm
*V* = 3131.1 (3) Å^3^	

### Data collection

**Table:**

Rigaku HG Saturn724+ (2x2 bin mode) diffractometer	17527 independent reflections
Radiation source: Enhance (Mo) X-ray Source	14543 reflections with *I* > 2σ(*I*)
Graphite monochromator	*R*_int_ = 0.131
ω scans	θ_max_ = 27.5°, θ_min_ = 2.1°
Absorption correction: multi-scan (*CrysAlis PRO*; Agilent, 2014)	*h* = −12→11
*T*_min_ = 0.82, *T*_max_ = 0.92	*k* = −19→17
29886 measured reflections	*l* = −29→29

### Refinement

**Table:**

Refinement on *F*^2^	0 restraints
Least-squares matrix: full	Hydrogen site location: inferred from neighbouring sites
*R*[*F*^2^ > 2σ(*F*^2^)] = 0.050	H-atom parameters constrained
*wR*(*F*^2^) = 0.125	*w* = 1/[σ^2^(*F*_o_^2^) + (0.0535*P*)^2^ + 12.5894*P*] where *P* = (*F*_o_^2^ + 2*F*_c_^2^)/3
*S* = 1.04	(Δ/σ)_max_ = 0.001
17491 reflections	Δρ_max_ = 0.77 e Å^−^^3^
758 parameters	Δρ_min_ = −0.61 e Å^−^^3^

### Special details

**Table:**

Geometry. All e.s.d.'s (except the e.s.d. in the dihedral angle between two l.s. planes) are estimated using the full covariance matrix. The cell e.s.d.'s are taken into account individually in the estimation of e.s.d.'s in distances, angles and torsion angles; correlations between e.s.d.'s in cell parameters are only used when they are defined by crystal symmetry. An approximate (isotropic) treatment of cell e.s.d.'s is used for estimating e.s.d.'s involving l.s. planes.

### Fractional atomic coordinates and isotropic or equivalent isotropic displacement parameters (Å^2^)

**Table:**

	*x*	*y*	*z*	*U*_iso_*/*U*_eq_	
C1	0.6368 (6)	0.2689 (4)	0.4096 (3)	0.0126 (12)	
C2	0.5367 (6)	0.2010 (4)	0.4103 (3)	0.0170 (13)	
H2	0.4851	0.2009	0.3760	0.020*	
C3	0.5112 (7)	0.1331 (4)	0.4609 (3)	0.0211 (14)	
H3	0.4426	0.0866	0.4606	0.025*	
C4	0.5834 (7)	0.1320 (4)	0.5111 (3)	0.0214 (14)	
H4	0.5643	0.0858	0.5458	0.026*	
C5	0.6856 (7)	0.1998 (4)	0.5106 (3)	0.0182 (13)	
H5	0.7381	0.1988	0.5448	0.022*	
C6	0.7111 (6)	0.2688 (4)	0.4603 (3)	0.0165 (13)	
H6	0.7789	0.3156	0.4606	0.020*	
C7	0.8425 (6)	0.3987 (4)	0.3433 (3)	0.0151 (13)	
C8	0.9474 (6)	0.3335 (4)	0.3461 (3)	0.0142 (12)	
H8	0.9233	0.2699	0.3549	0.017*	
C9	1.0872 (6)	0.3605 (4)	0.3363 (3)	0.0164 (13)	
H9	1.1584	0.3154	0.3383	0.020*	
C10	1.1232 (6)	0.4530 (4)	0.3234 (3)	0.0189 (14)	
H10	1.2189	0.4715	0.3158	0.023*	
C11	1.0188 (7)	0.5186 (4)	0.3217 (3)	0.0218 (15)	
H11	1.0436	0.5821	0.3138	0.026*	
C12	0.8791 (7)	0.4924 (4)	0.3314 (3)	0.0170 (13)	
H12	0.8082	0.5376	0.3300	0.020*	
C13	0.5496 (6)	0.4520 (4)	0.3611 (3)	0.0123 (12)	
C14	0.4866 (7)	0.4509 (4)	0.4183 (3)	0.0201 (14)	
H14	0.5010	0.4007	0.4498	0.024*	
C15	0.4018 (6)	0.5239 (4)	0.4295 (3)	0.0214 (14)	
H15	0.3575	0.5231	0.4686	0.026*	
C16	0.3821 (7)	0.5969 (4)	0.3842 (3)	0.0209 (14)	
H16	0.3252	0.6466	0.3924	0.025*	
C17	0.4437 (6)	0.5991 (4)	0.3269 (3)	0.0178 (13)	
H17	0.4288	0.6496	0.2956	0.021*	
C18	0.5283 (7)	0.5260 (4)	0.3153 (3)	0.0148 (13)	
H18	0.5715	0.5269	0.2760	0.018*	
C19	0.5986 (6)	0.5075 (4)	0.1554 (3)	0.0140 (12)	
C20	0.4564 (6)	0.5150 (4)	0.1688 (3)	0.0202 (13)	
H20	0.4010	0.4608	0.1821	0.024*	
C21	0.3928 (7)	0.5998 (4)	0.1632 (3)	0.0246 (14)	
H21	0.2944	0.6041	0.1715	0.030*	
C22	0.4749 (7)	0.6788 (4)	0.1453 (3)	0.0233 (14)	
H22	0.4330	0.7377	0.1423	0.028*	
C23	0.6150 (7)	0.6722 (4)	0.1322 (3)	0.0217 (13)	
H23	0.6701	0.7267	0.1199	0.026*	
C24	0.6789 (6)	0.5872 (3)	0.1366 (3)	0.0140 (11)	
H24	0.7769	0.5835	0.1267	0.017*	
C25	0.8566 (6)	0.4091 (4)	0.1397 (3)	0.0144 (12)	
C26	0.9058 (6)	0.4145 (4)	0.0801 (3)	0.0167 (12)	
H26	0.8408	0.4124	0.0506	0.020*	
C27	1.0477 (6)	0.4230 (4)	0.0626 (3)	0.0170 (12)	
H27	1.0795	0.4260	0.0215	0.020*	
C28	1.1434 (6)	0.4271 (4)	0.1056 (3)	0.0218 (13)	
H28	1.2410	0.4325	0.0940	0.026*	
C29	1.0952 (6)	0.4232 (4)	0.1657 (3)	0.0199 (13)	
H29	1.1599	0.4273	0.1951	0.024*	
C30	0.9531 (7)	0.4133 (4)	0.1826 (3)	0.0203 (14)	
H30	0.9210	0.4093	0.2238	0.024*	
C31	0.5948 (6)	0.3398 (4)	0.1085 (3)	0.0135 (11)	
C32	0.5816 (6)	0.2438 (4)	0.1186 (3)	0.0162 (12)	
H32	0.6138	0.2078	0.1540	0.019*	
C33	0.5207 (6)	0.2017 (4)	0.0762 (3)	0.0201 (13)	
H33	0.5117	0.1365	0.0827	0.024*	
C34	0.4735 (6)	0.2541 (4)	0.0249 (3)	0.0227 (13)	
H34	0.4313	0.2252	−0.0037	0.027*	
C35	0.4878 (7)	0.3488 (4)	0.0153 (3)	0.0276 (15)	
H35	0.4565	0.3850	−0.0202	0.033*	
C36	0.5474 (7)	0.3911 (4)	0.0574 (3)	0.0217 (13)	
H36	0.5557	0.4563	0.0508	0.026*	
C37	0.8919 (6)	0.7704 (4)	0.4118 (3)	0.0147 (12)	
C38	0.9933 (7)	0.7021 (4)	0.4114 (3)	0.0169 (13)	
H38	1.0563	0.7026	0.3768	0.020*	
C39	1.0014 (7)	0.6332 (4)	0.4620 (3)	0.0217 (14)	
H39	1.0704	0.5868	0.4622	0.026*	
C40	0.9087 (7)	0.6330 (4)	0.5117 (3)	0.0197 (14)	
H40	0.9133	0.5857	0.5460	0.024*	
C41	0.8091 (7)	0.7010 (4)	0.5121 (3)	0.0232 (15)	
H41	0.7464	0.7007	0.5468	0.028*	
C42	0.8011 (6)	0.7692 (4)	0.4622 (3)	0.0178 (13)	
H42	0.7324	0.8157	0.4625	0.021*	
C43	0.7131 (6)	0.9001 (4)	0.3442 (3)	0.0106 (11)	
C44	0.6065 (7)	0.8334 (4)	0.3476 (3)	0.0163 (13)	
H44	0.6275	0.7698	0.3573	0.020*	
C45	0.4721 (6)	0.8606 (4)	0.3369 (3)	0.0158 (13)	
H45	0.4002	0.8153	0.3395	0.019*	
C46	0.4393 (7)	0.9533 (4)	0.3223 (3)	0.0200 (14)	
H46	0.3461	0.9711	0.3141	0.024*	
C47	0.5421 (6)	1.0187 (4)	0.3198 (3)	0.0206 (14)	
H47	0.5200	1.0821	0.3103	0.025*	
C48	0.6786 (7)	0.9925 (4)	0.3311 (3)	0.0155 (13)	
H48	0.7490	1.0383	0.3299	0.019*	
C49	0.9967 (6)	0.9528 (4)	0.3630 (3)	0.0140 (12)	
C50	1.0347 (7)	1.0279 (4)	0.3177 (3)	0.0171 (13)	
H50	1.0064	1.0292	0.2781	0.021*	
C51	1.1122 (6)	1.0997 (4)	0.3295 (3)	0.0188 (13)	
H51	1.1342	1.1514	0.2986	0.023*	
C52	1.1586 (6)	1.0971 (4)	0.3865 (3)	0.0184 (13)	
H52	1.2144	1.1460	0.3946	0.022*	
C53	1.1226 (7)	1.0224 (4)	0.4316 (3)	0.0218 (14)	
H53	1.1527	1.0209	0.4709	0.026*	
C54	1.0439 (6)	0.9501 (4)	0.4201 (3)	0.0158 (13)	
H54	1.0218	0.8986	0.4511	0.019*	
C55	0.7747 (5)	0.9123 (4)	0.1395 (3)	0.0119 (11)	
C56	0.6601 (6)	0.9147 (4)	0.1809 (3)	0.0147 (12)	
H56	0.6735	0.9084	0.2226	0.018*	
C57	0.5278 (7)	0.9263 (4)	0.1613 (3)	0.0254 (15)	
H57	0.4499	0.9276	0.1898	0.031*	
C58	0.5064 (6)	0.9362 (4)	0.1014 (3)	0.0208 (13)	
H58	0.4141	0.9444	0.0885	0.025*	
C59	0.6189 (6)	0.9342 (4)	0.0596 (3)	0.0206 (13)	
H59	0.6040	0.9407	0.0180	0.025*	
C60	0.7534 (6)	0.9228 (4)	0.0784 (3)	0.0148 (12)	
H60	0.8310	0.9222	0.0497	0.018*	
C61	1.0405 (6)	0.8386 (4)	0.1112 (3)	0.0142 (11)	
C62	1.0293 (6)	0.7432 (4)	0.1160 (3)	0.0185 (12)	
H62	0.9773	0.7081	0.1498	0.022*	
C63	1.0929 (7)	0.6993 (4)	0.0722 (3)	0.0242 (14)	
H63	1.0822	0.6346	0.0755	0.029*	
C64	1.1720 (7)	0.7492 (4)	0.0238 (3)	0.0253 (14)	
H64	1.2165	0.7188	−0.0060	0.030*	
C65	1.1863 (6)	0.8440 (4)	0.0187 (3)	0.0210 (13)	
H65	1.2414	0.8783	−0.0145	0.025*	
C66	1.1208 (6)	0.8884 (4)	0.0617 (3)	0.0180 (12)	
H66	1.1302	0.9533	0.0577	0.022*	
C67	1.0306 (6)	1.0063 (4)	0.1571 (2)	0.0109 (11)	
C68	0.9670 (7)	1.0857 (4)	0.1310 (3)	0.0241 (14)	
H68	0.8750	1.0826	0.1182	0.029*	
C69	1.0363 (7)	1.1706 (4)	0.1233 (3)	0.0318 (16)	
H69	0.9930	1.2248	0.1041	0.038*	
C70	1.1680 (7)	1.1754 (4)	0.1440 (3)	0.0232 (14)	
H70	1.2152	1.2331	0.1393	0.028*	
C71	1.2305 (6)	1.0970 (4)	0.1711 (3)	0.0212 (13)	
H71	1.3206	1.1007	0.1857	0.025*	
C72	1.1639 (6)	1.0126 (4)	0.1774 (3)	0.0160 (12)	
H72	1.2090	0.9585	0.1958	0.019*	
Br1	0.38942 (6)	0.27817 (4)	0.25911 (3)	0.01690 (14)	
Br2	1.19526 (6)	0.77398 (4)	0.26025 (3)	0.01775 (14)	
N1	0.7649 (5)	0.2230 (3)	0.2556 (2)	0.0154 (10)	
N2	0.8214 (5)	0.7256 (3)	0.2586 (2)	0.0164 (11)	
Ni1	0.63731 (7)	0.30337 (5)	0.25759 (3)	0.01108 (16)	
Ni2	0.94837 (7)	0.80352 (5)	0.25946 (3)	0.01098 (16)	
O1	0.8806 (5)	0.2047 (3)	0.2489 (2)	0.0320 (12)	
O2	0.7053 (5)	0.7066 (3)	0.2545 (2)	0.0282 (11)	
P1	0.66202 (17)	0.35864 (10)	0.34305 (7)	0.0110 (3)	
P2	0.67266 (15)	0.39238 (10)	0.16627 (7)	0.0101 (3)	
P3	0.89261 (17)	0.86003 (10)	0.34469 (7)	0.0110 (3)	
P4	0.94851 (15)	0.89306 (10)	0.16829 (7)	0.0114 (3)	

### Atomic displacement parameters (Å^2^)

**Table:**

	*U*^11^	*U*^22^	*U*^33^	*U*^12^	*U*^13^	*U*^23^
C1	0.015 (3)	0.013 (3)	0.010 (3)	0.005 (2)	−0.003 (2)	0.000 (2)
C2	0.015 (3)	0.021 (3)	0.016 (3)	−0.002 (2)	0.001 (3)	−0.007 (3)
C3	0.026 (4)	0.019 (3)	0.018 (3)	−0.002 (3)	0.007 (3)	−0.004 (3)
C4	0.027 (4)	0.016 (3)	0.018 (3)	0.004 (3)	0.006 (3)	0.002 (3)
C5	0.023 (3)	0.023 (3)	0.008 (3)	0.003 (3)	−0.003 (3)	0.000 (3)
C6	0.014 (3)	0.014 (3)	0.021 (3)	0.002 (2)	−0.001 (3)	−0.004 (3)
C7	0.014 (3)	0.020 (3)	0.013 (3)	−0.001 (2)	−0.001 (2)	−0.006 (3)
C8	0.013 (3)	0.015 (3)	0.015 (3)	0.001 (2)	0.002 (3)	−0.006 (2)
C9	0.006 (3)	0.025 (3)	0.018 (3)	0.004 (2)	0.001 (3)	−0.006 (3)
C10	0.008 (3)	0.030 (3)	0.019 (3)	−0.010 (3)	0.001 (3)	−0.007 (3)
C11	0.024 (4)	0.017 (3)	0.024 (4)	−0.007 (3)	0.001 (3)	−0.003 (3)
C12	0.012 (3)	0.018 (3)	0.022 (4)	−0.003 (3)	0.000 (3)	−0.008 (3)
C13	0.007 (3)	0.015 (3)	0.015 (3)	−0.003 (2)	0.005 (2)	−0.006 (2)
C14	0.024 (3)	0.018 (3)	0.017 (3)	0.000 (3)	−0.001 (3)	0.001 (3)
C15	0.016 (3)	0.026 (3)	0.022 (4)	0.002 (3)	0.006 (3)	−0.006 (3)
C16	0.020 (3)	0.015 (3)	0.029 (4)	0.002 (2)	−0.001 (3)	−0.007 (3)
C17	0.013 (3)	0.016 (3)	0.024 (3)	0.002 (2)	−0.001 (3)	−0.002 (3)
C18	0.014 (3)	0.017 (3)	0.011 (3)	−0.002 (3)	0.002 (3)	0.001 (2)
C19	0.018 (3)	0.013 (3)	0.013 (3)	0.004 (2)	−0.006 (2)	−0.003 (2)
C20	0.020 (3)	0.018 (3)	0.022 (3)	−0.003 (2)	−0.003 (3)	−0.001 (2)
C21	0.024 (3)	0.027 (3)	0.023 (3)	0.012 (3)	−0.005 (3)	−0.003 (3)
C22	0.037 (4)	0.014 (3)	0.022 (3)	0.012 (2)	−0.013 (3)	−0.007 (2)
C23	0.032 (3)	0.011 (3)	0.024 (3)	−0.001 (2)	−0.011 (3)	−0.003 (2)
C24	0.015 (3)	0.011 (3)	0.017 (3)	−0.002 (2)	−0.007 (2)	−0.005 (2)
C25	0.019 (3)	0.010 (3)	0.014 (3)	−0.004 (2)	−0.002 (2)	0.000 (2)
C26	0.021 (3)	0.011 (3)	0.018 (3)	0.000 (2)	−0.006 (2)	−0.001 (2)
C27	0.017 (3)	0.016 (3)	0.017 (3)	−0.002 (2)	0.004 (2)	−0.003 (2)
C28	0.010 (3)	0.026 (3)	0.029 (4)	0.002 (2)	0.002 (3)	−0.005 (3)
C29	0.006 (3)	0.034 (4)	0.017 (3)	−0.002 (2)	0.000 (2)	0.001 (3)
C30	0.025 (3)	0.021 (3)	0.014 (3)	−0.004 (3)	0.000 (3)	−0.002 (3)
C31	0.012 (3)	0.016 (3)	0.014 (3)	−0.006 (2)	0.002 (2)	−0.008 (2)
C32	0.021 (3)	0.013 (3)	0.013 (3)	0.000 (2)	0.003 (2)	−0.002 (2)
C33	0.030 (3)	0.013 (3)	0.018 (3)	−0.006 (2)	0.004 (2)	−0.007 (2)
C34	0.027 (3)	0.021 (3)	0.023 (3)	−0.003 (2)	−0.003 (3)	−0.011 (3)
C35	0.039 (4)	0.019 (3)	0.027 (3)	0.009 (3)	−0.016 (3)	−0.004 (3)
C36	0.035 (4)	0.012 (3)	0.019 (3)	0.004 (2)	−0.011 (3)	−0.002 (2)
C37	0.017 (3)	0.012 (3)	0.017 (3)	−0.002 (2)	−0.011 (3)	−0.003 (2)
C38	0.024 (3)	0.015 (3)	0.011 (3)	−0.001 (2)	−0.002 (3)	0.000 (2)
C39	0.028 (4)	0.013 (3)	0.023 (4)	0.007 (3)	−0.002 (3)	−0.003 (3)
C40	0.033 (4)	0.015 (3)	0.010 (3)	−0.005 (3)	−0.003 (3)	0.001 (2)
C41	0.028 (4)	0.022 (3)	0.020 (4)	−0.001 (3)	0.002 (3)	−0.007 (3)
C42	0.020 (3)	0.018 (3)	0.015 (3)	0.002 (2)	0.000 (3)	−0.004 (3)
C43	0.010 (3)	0.012 (3)	0.009 (3)	0.002 (2)	0.000 (2)	−0.002 (2)
C44	0.014 (3)	0.021 (3)	0.012 (3)	−0.002 (2)	0.004 (3)	−0.002 (3)
C45	0.018 (3)	0.016 (3)	0.014 (3)	−0.001 (2)	0.001 (3)	−0.007 (2)
C46	0.015 (3)	0.028 (4)	0.017 (3)	0.003 (3)	−0.001 (3)	−0.006 (3)
C47	0.017 (3)	0.020 (3)	0.026 (4)	0.009 (3)	−0.002 (3)	−0.007 (3)
C48	0.015 (3)	0.018 (3)	0.013 (3)	−0.004 (3)	0.000 (3)	−0.001 (3)
C49	0.010 (3)	0.013 (3)	0.018 (3)	−0.003 (2)	0.000 (3)	−0.002 (2)
C50	0.018 (3)	0.019 (3)	0.016 (3)	0.001 (3)	−0.004 (3)	−0.006 (3)
C51	0.014 (3)	0.016 (3)	0.025 (4)	0.001 (2)	0.002 (3)	0.001 (3)
C52	0.011 (3)	0.020 (3)	0.026 (4)	−0.004 (2)	−0.004 (3)	−0.008 (3)
C53	0.026 (4)	0.024 (3)	0.019 (3)	0.000 (3)	−0.005 (3)	−0.009 (3)
C54	0.016 (3)	0.016 (3)	0.016 (3)	−0.005 (2)	−0.003 (3)	−0.003 (3)
C55	0.006 (2)	0.011 (3)	0.019 (3)	−0.0013 (19)	−0.002 (2)	−0.004 (2)
C56	0.004 (3)	0.026 (3)	0.013 (3)	0.003 (2)	−0.002 (2)	0.000 (2)
C57	0.019 (3)	0.029 (3)	0.026 (4)	0.003 (3)	0.002 (3)	−0.002 (3)
C58	0.016 (3)	0.012 (3)	0.033 (4)	0.001 (2)	−0.012 (3)	0.003 (2)
C59	0.023 (3)	0.016 (3)	0.024 (3)	−0.002 (2)	−0.010 (3)	−0.004 (2)
C60	0.014 (3)	0.014 (3)	0.015 (3)	0.002 (2)	−0.002 (2)	−0.001 (2)
C61	0.011 (3)	0.017 (3)	0.014 (3)	−0.002 (2)	−0.003 (2)	0.001 (2)
C62	0.020 (3)	0.018 (3)	0.017 (3)	−0.003 (2)	0.003 (2)	−0.005 (2)
C63	0.029 (3)	0.018 (3)	0.029 (3)	0.003 (2)	−0.004 (3)	−0.011 (3)
C64	0.026 (3)	0.026 (3)	0.027 (4)	0.000 (3)	0.006 (3)	−0.015 (3)
C65	0.024 (3)	0.023 (3)	0.017 (3)	−0.002 (2)	0.006 (3)	−0.007 (2)
C66	0.015 (3)	0.016 (3)	0.023 (3)	0.000 (2)	0.003 (2)	−0.005 (2)
C67	0.011 (3)	0.011 (2)	0.011 (3)	0.002 (2)	0.003 (2)	−0.004 (2)
C68	0.018 (3)	0.020 (3)	0.036 (4)	0.005 (2)	−0.007 (3)	−0.006 (3)
C69	0.034 (4)	0.015 (3)	0.045 (4)	0.004 (3)	−0.007 (3)	−0.001 (3)
C70	0.023 (3)	0.015 (3)	0.031 (4)	−0.008 (2)	0.004 (3)	−0.003 (3)
C71	0.015 (3)	0.025 (3)	0.024 (3)	−0.007 (2)	0.002 (3)	−0.005 (3)
C72	0.009 (3)	0.016 (3)	0.022 (3)	−0.003 (2)	0.001 (2)	0.000 (2)
Br1	0.0096 (3)	0.0202 (3)	0.0202 (3)	−0.0030 (2)	0.0005 (3)	−0.0024 (3)
Br2	0.0113 (3)	0.0204 (3)	0.0206 (3)	0.0026 (2)	−0.0021 (3)	−0.0008 (3)
N1	0.015 (3)	0.019 (3)	0.013 (3)	0.004 (2)	−0.003 (2)	−0.005 (2)
N2	0.015 (3)	0.022 (3)	0.013 (3)	0.000 (2)	−0.004 (2)	−0.004 (2)
Ni1	0.0099 (4)	0.0111 (3)	0.0119 (4)	0.0003 (3)	−0.0001 (3)	−0.0016 (3)
Ni2	0.0105 (4)	0.0106 (3)	0.0118 (4)	0.0008 (3)	−0.0007 (3)	−0.0017 (3)
O1	0.021 (3)	0.035 (3)	0.039 (3)	0.017 (2)	−0.001 (2)	−0.004 (2)
O2	0.024 (3)	0.031 (3)	0.030 (3)	−0.011 (2)	0.002 (2)	−0.008 (2)
P1	0.0090 (7)	0.0102 (7)	0.0131 (8)	−0.0001 (6)	0.0004 (7)	−0.0011 (6)
P2	0.0072 (7)	0.0112 (7)	0.0116 (7)	−0.0004 (5)	−0.0004 (6)	−0.0012 (6)
P3	0.0098 (7)	0.0116 (7)	0.0117 (8)	0.0008 (6)	−0.0010 (6)	−0.0026 (6)
P4	0.0100 (7)	0.0121 (7)	0.0116 (7)	0.0007 (5)	0.0006 (6)	−0.0017 (6)

### Geometric parameters (Å, º)

**Table:**

C1—C2	1.384 (8)	C39—C40	1.379 (9)
C1—C6	1.392 (8)	C39—H39	0.9500
C1—P1	1.820 (6)	C40—C41	1.384 (9)
C2—C3	1.385 (9)	C40—H40	0.9500
C2—H2	0.9500	C41—C42	1.379 (9)
C3—C4	1.370 (9)	C41—H41	0.9500
C3—H3	0.9500	C42—H42	0.9500
C4—C5	1.398 (9)	C43—C48	1.388 (8)
C4—H4	0.9500	C43—C44	1.406 (8)
C5—C6	1.391 (8)	C43—P3	1.815 (6)
C5—H5	0.9500	C44—C45	1.371 (8)
C6—H6	0.9500	C44—H44	0.9500
C7—C8	1.387 (8)	C45—C46	1.391 (8)
C7—C12	1.401 (8)	C45—H45	0.9500
C7—P1	1.825 (6)	C46—C47	1.370 (9)
C8—C9	1.385 (8)	C46—H46	0.9500
C8—H8	0.9500	C47—C48	1.389 (9)
C9—C10	1.382 (8)	C47—H47	0.9500
C9—H9	0.9500	C48—H48	0.9500
C10—C11	1.388 (9)	C49—C54	1.392 (8)
C10—H10	0.9500	C49—C50	1.397 (8)
C11—C12	1.381 (9)	C49—P3	1.818 (6)
C11—H11	0.9500	C50—C51	1.371 (8)
C12—H12	0.9500	C50—H50	0.9500
C13—C14	1.382 (8)	C51—C52	1.388 (9)
C13—C18	1.391 (8)	C51—H51	0.9500
C13—P1	1.832 (6)	C52—C53	1.386 (9)
C14—C15	1.397 (8)	C52—H52	0.9500
C14—H14	0.9500	C53—C54	1.379 (8)
C15—C16	1.370 (9)	C53—H53	0.9500
C15—H15	0.9500	C54—H54	0.9500
C16—C17	1.378 (9)	C55—C56	1.391 (7)
C16—H16	0.9500	C55—C60	1.393 (8)
C17—C18	1.399 (8)	C55—P4	1.832 (5)
C17—H17	0.9500	C56—C57	1.371 (8)
C18—H18	0.9500	C56—H56	0.9500
C19—C20	1.379 (8)	C57—C58	1.367 (9)
C19—C24	1.390 (8)	C57—H57	0.9500
C19—P2	1.823 (5)	C58—C59	1.382 (9)
C20—C21	1.382 (8)	C58—H58	0.9500
C20—H20	0.9500	C59—C60	1.384 (8)
C21—C22	1.388 (9)	C59—H59	0.9500
C21—H21	0.9500	C60—H60	0.9500
C22—C23	1.357 (9)	C61—C62	1.397 (8)
C22—H22	0.9500	C61—C66	1.401 (8)
C23—C24	1.387 (8)	C61—P4	1.814 (6)
C23—H23	0.9500	C62—C63	1.382 (8)
C24—H24	0.9500	C62—H62	0.9500
C25—C26	1.381 (8)	C63—C64	1.380 (9)
C25—C30	1.396 (8)	C63—H63	0.9500
C25—P2	1.818 (6)	C64—C65	1.390 (8)
C26—C27	1.384 (8)	C64—H64	0.9500
C26—H26	0.9500	C65—C66	1.381 (7)
C27—C28	1.392 (8)	C65—H65	0.9500
C27—H27	0.9500	C66—H66	0.9500
C28—C29	1.394 (8)	C67—C68	1.377 (8)
C28—H28	0.9500	C67—C72	1.395 (8)
C29—C30	1.384 (8)	C67—P4	1.816 (5)
C29—H29	0.9500	C68—C69	1.396 (9)
C30—H30	0.9500	C68—H68	0.9500
C31—C36	1.371 (8)	C69—C70	1.381 (9)
C31—C32	1.399 (7)	C69—H69	0.9500
C31—P2	1.835 (5)	C70—C71	1.368 (9)
C32—C33	1.393 (8)	C70—H70	0.9500
C32—H32	0.9500	C71—C72	1.380 (8)
C33—C34	1.380 (9)	C71—H71	0.9500
C33—H33	0.9500	C72—H72	0.9500
C34—C35	1.382 (8)	Br1—Ni1	2.3956 (9)
C34—H34	0.9500	Br2—Ni2	2.4002 (9)
C35—C36	1.383 (8)	N1—O1	1.144 (6)
C35—H35	0.9500	N1—Ni1	1.705 (5)
C36—H36	0.9500	N2—O2	1.159 (6)
C37—C42	1.377 (8)	N2—Ni2	1.678 (5)
C37—C38	1.400 (8)	Ni1—P2	2.2434 (16)
C37—P3	1.826 (6)	Ni1—P1	2.2553 (17)
C38—C39	1.395 (8)	Ni2—P4	2.2412 (17)
C38—H38	0.9500	Ni2—P3	2.2615 (16)
			
C2—C1—C6	119.3 (6)	C48—C43—P3	123.1 (4)
C2—C1—P1	118.6 (5)	C44—C43—P3	117.2 (4)
C6—C1—P1	122.0 (4)	C45—C44—C43	119.7 (6)
C1—C2—C3	120.4 (6)	C45—C44—H44	120.2
C1—C2—H2	119.8	C43—C44—H44	120.2
C3—C2—H2	119.8	C44—C45—C46	121.1 (6)
C4—C3—C2	120.9 (6)	C44—C45—H45	119.5
C4—C3—H3	119.5	C46—C45—H45	119.5
C2—C3—H3	119.5	C47—C46—C45	119.5 (6)
C3—C4—C5	119.0 (6)	C47—C46—H46	120.2
C3—C4—H4	120.5	C45—C46—H46	120.2
C5—C4—H4	120.5	C46—C47—C48	120.2 (6)
C6—C5—C4	120.5 (6)	C46—C47—H47	119.9
C6—C5—H5	119.8	C48—C47—H47	119.9
C4—C5—H5	119.8	C43—C48—C47	120.7 (6)
C5—C6—C1	119.8 (6)	C43—C48—H48	119.7
C5—C6—H6	120.1	C47—C48—H48	119.7
C1—C6—H6	120.1	C54—C49—C50	118.9 (5)
C8—C7—C12	119.4 (6)	C54—C49—P3	122.3 (5)
C8—C7—P1	117.7 (4)	C50—C49—P3	118.8 (5)
C12—C7—P1	122.2 (5)	C51—C50—C49	120.9 (6)
C9—C8—C7	120.4 (6)	C51—C50—H50	119.6
C9—C8—H8	119.8	C49—C50—H50	119.6
C7—C8—H8	119.8	C50—C51—C52	120.1 (6)
C10—C9—C8	120.2 (6)	C50—C51—H51	119.9
C10—C9—H9	119.9	C52—C51—H51	120.0
C8—C9—H9	119.9	C53—C52—C51	119.3 (6)
C9—C10—C11	119.7 (5)	C53—C52—H52	120.3
C9—C10—H10	120.2	C51—C52—H52	120.3
C11—C10—H10	120.2	C54—C53—C52	120.9 (6)
C12—C11—C10	120.6 (6)	C54—C53—H53	119.6
C12—C11—H11	119.7	C52—C53—H53	119.6
C10—C11—H11	119.7	C53—C54—C49	119.9 (6)
C11—C12—C7	119.7 (6)	C53—C54—H54	120.0
C11—C12—H12	120.1	C49—C54—H54	120.0
C7—C12—H12	120.1	C56—C55—C60	119.3 (5)
C14—C13—C18	119.8 (5)	C56—C55—P4	118.2 (4)
C14—C13—P1	122.2 (5)	C60—C55—P4	122.5 (4)
C18—C13—P1	118.1 (5)	C57—C56—C55	120.0 (6)
C13—C14—C15	119.7 (6)	C57—C56—H56	120.0
C13—C14—H14	120.2	C55—C56—H56	120.0
C15—C14—H14	120.2	C58—C57—C56	120.9 (6)
C16—C15—C14	120.2 (6)	C58—C57—H57	119.6
C16—C15—H15	119.9	C56—C57—H57	119.6
C14—C15—H15	119.9	C57—C58—C59	120.0 (6)
C15—C16—C17	120.9 (6)	C57—C58—H58	120.0
C15—C16—H16	119.5	C59—C58—H58	120.0
C17—C16—H16	119.5	C58—C59—C60	120.0 (6)
C16—C17—C18	119.2 (6)	C58—C59—H59	120.0
C16—C17—H17	120.4	C60—C59—H59	120.0
C18—C17—H17	120.4	C59—C60—C55	119.8 (6)
C13—C18—C17	120.3 (6)	C59—C60—H60	120.1
C13—C18—H18	119.9	C55—C60—H60	120.1
C17—C18—H18	119.9	C62—C61—C66	118.4 (5)
C20—C19—C24	119.0 (5)	C62—C61—P4	118.9 (4)
C20—C19—P2	117.8 (5)	C66—C61—P4	122.7 (4)
C24—C19—P2	123.2 (4)	C63—C62—C61	120.8 (6)
C19—C20—C21	121.3 (6)	C63—C62—H62	119.6
C19—C20—H20	119.4	C61—C62—H62	119.6
C21—C20—H20	119.4	C64—C63—C62	120.2 (5)
C20—C21—C22	119.0 (6)	C64—C63—H63	119.9
C20—C21—H21	120.5	C62—C63—H63	119.9
C22—C21—H21	120.5	C63—C64—C65	119.9 (5)
C23—C22—C21	120.1 (5)	C63—C64—H64	120.0
C23—C22—H22	119.9	C65—C64—H64	120.0
C21—C22—H22	119.9	C66—C65—C64	120.2 (6)
C22—C23—C24	121.1 (6)	C66—C65—H65	119.9
C22—C23—H23	119.4	C64—C65—H65	119.9
C24—C23—H23	119.4	C65—C66—C61	120.5 (5)
C23—C24—C19	119.4 (5)	C65—C66—H66	119.8
C23—C24—H24	120.3	C61—C66—H66	119.8
C19—C24—H24	120.3	C68—C67—C72	118.8 (5)
C26—C25—C30	118.7 (6)	C68—C67—P4	123.5 (4)
C26—C25—P2	123.7 (5)	C72—C67—P4	117.7 (4)
C30—C25—P2	117.6 (5)	C67—C68—C69	120.7 (6)
C25—C26—C27	121.5 (6)	C67—C68—H68	119.7
C25—C26—H26	119.3	C69—C68—H68	119.7
C27—C26—H26	119.3	C70—C69—C68	119.6 (6)
C26—C27—C28	119.6 (6)	C70—C69—H69	120.2
C26—C27—H27	120.2	C68—C69—H69	120.2
C28—C27—H27	120.2	C71—C70—C69	120.0 (6)
C27—C28—C29	119.6 (5)	C71—C70—H70	120.0
C27—C28—H28	120.2	C69—C70—H70	120.0
C29—C28—H28	120.2	C70—C71—C72	120.6 (6)
C30—C29—C28	120.0 (6)	C70—C71—H71	119.7
C30—C29—H29	120.0	C72—C71—H71	119.7
C28—C29—H29	120.0	C71—C72—C67	120.3 (6)
C29—C30—C25	120.6 (6)	C71—C72—H72	119.8
C29—C30—H30	119.7	C67—C72—H72	119.8
C25—C30—H30	119.7	O1—N1—Ni1	150.2 (5)
C36—C31—C32	119.8 (5)	O2—N2—Ni2	151.2 (5)
C36—C31—P2	122.4 (4)	N1—Ni1—P2	102.30 (18)
C32—C31—P2	117.8 (4)	N1—Ni1—P1	105.22 (17)
C33—C32—C31	119.4 (5)	P2—Ni1—P1	121.86 (6)
C33—C32—H32	120.3	N1—Ni1—Br1	126.70 (17)
C31—C32—H32	120.3	P2—Ni1—Br1	99.18 (5)
C34—C33—C32	120.3 (5)	P1—Ni1—Br1	103.48 (5)
C34—C33—H33	119.9	N2—Ni2—P4	103.55 (18)
C32—C33—H33	119.9	N2—Ni2—P3	104.09 (18)
C33—C34—C35	119.9 (6)	P4—Ni2—P3	121.40 (6)
C33—C34—H34	120.1	N2—Ni2—Br2	126.09 (17)
C35—C34—H34	120.1	P4—Ni2—Br2	98.57 (5)
C34—C35—C36	120.1 (6)	P3—Ni2—Br2	104.93 (5)
C34—C35—H35	119.9	C1—P1—C7	104.7 (3)
C36—C35—H35	119.9	C1—P1—C13	104.0 (3)
C31—C36—C35	120.6 (5)	C7—P1—C13	106.3 (3)
C31—C36—H36	119.7	C1—P1—Ni1	111.76 (19)
C35—C36—H36	119.7	C7—P1—Ni1	109.2 (2)
C42—C37—C38	119.8 (6)	C13—P1—Ni1	119.7 (2)
C42—C37—P3	123.5 (5)	C25—P2—C19	105.2 (3)
C38—C37—P3	116.7 (5)	C25—P2—C31	104.4 (3)
C39—C38—C37	119.7 (6)	C19—P2—C31	103.0 (3)
C39—C38—H38	120.2	C25—P2—Ni1	113.80 (19)
C37—C38—H38	120.2	C19—P2—Ni1	118.0 (2)
C40—C39—C38	119.5 (6)	C31—P2—Ni1	111.10 (19)
C40—C39—H39	120.2	C43—P3—C49	106.1 (3)
C38—C39—H39	120.2	C43—P3—C37	104.2 (3)
C39—C40—C41	120.6 (6)	C49—P3—C37	104.1 (3)
C39—C40—H40	119.7	C43—P3—Ni2	109.13 (19)
C41—C40—H40	119.7	C49—P3—Ni2	120.2 (2)
C42—C41—C40	119.9 (6)	C37—P3—Ni2	111.81 (19)
C42—C41—H41	120.0	C61—P4—C67	103.5 (2)
C40—C41—H41	120.0	C61—P4—C55	102.2 (3)
C37—C42—C41	120.5 (6)	C67—P4—C55	105.4 (2)
C37—C42—H42	119.8	C61—P4—Ni2	111.01 (19)
C41—C42—H42	119.8	C67—P4—Ni2	118.49 (19)
C48—C43—C44	118.8 (5)	C55—P4—Ni2	114.53 (19)
			
C6—C1—C2—C3	−0.5 (9)	P4—C61—C66—C65	−178.6 (5)
P1—C1—C2—C3	−178.0 (5)	C72—C67—C68—C69	2.1 (9)
C1—C2—C3—C4	0.4 (9)	P4—C67—C68—C69	−179.6 (5)
C2—C3—C4—C5	−1.0 (9)	C67—C68—C69—C70	−2.2 (10)
C3—C4—C5—C6	1.6 (9)	C68—C69—C70—C71	0.7 (10)
C4—C5—C6—C1	−1.7 (9)	C69—C70—C71—C72	0.9 (10)
C2—C1—C6—C5	1.1 (8)	C70—C71—C72—C67	−1.0 (9)
P1—C1—C6—C5	178.5 (4)	C68—C67—C72—C71	−0.5 (9)
C12—C7—C8—C9	−1.2 (9)	P4—C67—C72—C71	−178.9 (5)
P1—C7—C8—C9	169.0 (5)	O1—N1—Ni1—P2	55.9 (10)
C7—C8—C9—C10	0.0 (10)	O1—N1—Ni1—P1	−72.4 (10)
C8—C9—C10—C11	1.3 (10)	O1—N1—Ni1—Br1	167.4 (9)
C9—C10—C11—C12	−1.4 (10)	O2—N2—Ni2—P4	−60.5 (10)
C10—C11—C12—C7	0.2 (10)	O2—N2—Ni2—P3	67.3 (10)
C8—C7—C12—C11	1.1 (9)	O2—N2—Ni2—Br2	−171.9 (9)
P1—C7—C12—C11	−168.7 (5)	C2—C1—P1—C7	−154.5 (5)
C18—C13—C14—C15	0.3 (9)	C6—C1—P1—C7	28.0 (5)
P1—C13—C14—C15	−179.9 (5)	C2—C1—P1—C13	94.1 (5)
C13—C14—C15—C16	−0.7 (9)	C6—C1—P1—C13	−83.4 (5)
C14—C15—C16—C17	0.9 (10)	C2—C1—P1—Ni1	−36.4 (5)
C15—C16—C17—C18	−0.6 (9)	C6—C1—P1—Ni1	146.1 (4)
C14—C13—C18—C17	0.0 (9)	C8—C7—P1—C1	54.2 (5)
P1—C13—C18—C17	−179.8 (5)	C12—C7—P1—C1	−135.9 (5)
C16—C17—C18—C13	0.2 (9)	C8—C7—P1—C13	163.9 (5)
C24—C19—C20—C21	0.6 (9)	C12—C7—P1—C13	−26.2 (6)
P2—C19—C20—C21	178.3 (5)	C8—C7—P1—Ni1	−65.6 (5)
C19—C20—C21—C22	−1.7 (10)	C12—C7—P1—Ni1	104.3 (5)
C20—C21—C22—C23	1.5 (9)	C14—C13—P1—C1	10.0 (6)
C21—C22—C23—C24	−0.2 (10)	C18—C13—P1—C1	−170.2 (5)
C22—C23—C24—C19	−0.9 (9)	C14—C13—P1—C7	−100.2 (5)
C20—C19—C24—C23	0.7 (9)	C18—C13—P1—C7	79.6 (5)
P2—C19—C24—C23	−176.9 (4)	C14—C13—P1—Ni1	135.6 (5)
C30—C25—C26—C27	0.7 (8)	C18—C13—P1—Ni1	−44.6 (5)
P2—C25—C26—C27	−176.9 (4)	C26—C25—P2—C19	−85.7 (5)
C25—C26—C27—C28	−0.6 (8)	C30—C25—P2—C19	96.6 (5)
C26—C27—C28—C29	−0.4 (9)	C26—C25—P2—C31	22.4 (6)
C27—C28—C29—C30	1.4 (9)	C30—C25—P2—C31	−155.3 (4)
C28—C29—C30—C25	−1.4 (10)	C26—C25—P2—Ni1	143.6 (4)
C26—C25—C30—C29	0.3 (9)	C30—C25—P2—Ni1	−34.0 (5)
P2—C25—C30—C29	178.1 (5)	C20—C19—P2—C25	176.3 (5)
C36—C31—C32—C33	0.2 (9)	C24—C19—P2—C25	−6.0 (6)
P2—C31—C32—C33	178.9 (4)	C20—C19—P2—C31	67.2 (5)
C31—C32—C33—C34	−0.2 (9)	C24—C19—P2—C31	−115.1 (5)
C32—C33—C34—C35	0.6 (9)	C20—C19—P2—Ni1	−55.5 (5)
C33—C34—C35—C36	−0.9 (10)	C24—C19—P2—Ni1	122.1 (5)
C32—C31—C36—C35	−0.5 (10)	C36—C31—P2—C25	−85.6 (5)
P2—C31—C36—C35	−179.2 (5)	C32—C31—P2—C25	95.7 (5)
C34—C35—C36—C31	0.9 (10)	C36—C31—P2—C19	24.1 (6)
C42—C37—C38—C39	0.0 (9)	C32—C31—P2—C19	−154.5 (5)
P3—C37—C38—C39	178.0 (5)	C36—C31—P2—Ni1	151.4 (5)
C37—C38—C39—C40	0.5 (9)	C32—C31—P2—Ni1	−27.3 (5)
C38—C39—C40—C41	−0.8 (10)	C48—C43—P3—C49	27.6 (6)
C39—C40—C41—C42	0.7 (10)	C44—C43—P3—C49	−163.0 (5)
C38—C37—C42—C41	−0.2 (9)	C48—C43—P3—C37	137.1 (5)
P3—C37—C42—C41	−177.9 (5)	C44—C43—P3—C37	−53.5 (5)
C40—C41—C42—C37	−0.2 (9)	C48—C43—P3—Ni2	−103.3 (5)
C48—C43—C44—C45	1.3 (9)	C44—C43—P3—Ni2	66.1 (5)
P3—C43—C44—C45	−168.6 (5)	C54—C49—P3—C43	103.1 (5)
C43—C44—C45—C46	0.4 (9)	C50—C49—P3—C43	−79.2 (5)
C44—C45—C46—C47	−1.4 (10)	C54—C49—P3—C37	−6.6 (6)
C45—C46—C47—C48	0.7 (10)	C50—C49—P3—C37	171.2 (5)
C44—C43—C48—C47	−1.9 (9)	C54—C49—P3—Ni2	−132.7 (5)
P3—C43—C48—C47	167.3 (5)	C50—C49—P3—Ni2	45.0 (6)
C46—C47—C48—C43	0.9 (10)	C42—C37—P3—C43	−28.2 (6)
C54—C49—C50—C51	−2.7 (9)	C38—C37—P3—C43	154.0 (4)
P3—C49—C50—C51	179.5 (5)	C42—C37—P3—C49	82.8 (5)
C49—C50—C51—C52	2.4 (9)	C38—C37—P3—C49	−95.0 (5)
C50—C51—C52—C53	−1.7 (9)	C42—C37—P3—Ni2	−145.9 (4)
C51—C52—C53—C54	1.2 (9)	C38—C37—P3—Ni2	36.3 (5)
C52—C53—C54—C49	−1.5 (9)	C62—C61—P4—C67	164.1 (5)
C50—C49—C54—C53	2.2 (9)	C66—C61—P4—C67	−16.9 (6)
P3—C49—C54—C53	179.9 (5)	C62—C61—P4—C55	−86.5 (5)
C60—C55—C56—C57	0.8 (9)	C66—C61—P4—C55	92.4 (5)
P4—C55—C56—C57	−178.2 (5)	C62—C61—P4—Ni2	36.0 (5)
C55—C56—C57—C58	−0.4 (9)	C66—C61—P4—Ni2	−145.0 (4)
C56—C57—C58—C59	0.2 (9)	C68—C67—P4—C61	106.1 (5)
C57—C58—C59—C60	−0.4 (9)	C72—C67—P4—C61	−75.6 (5)
C58—C59—C60—C55	0.7 (8)	C68—C67—P4—C55	−0.8 (6)
C56—C55—C60—C59	−0.9 (8)	C72—C67—P4—C55	177.5 (4)
P4—C55—C60—C59	178.0 (4)	C68—C67—P4—Ni2	−130.6 (5)
C66—C61—C62—C63	−1.7 (9)	C72—C67—P4—Ni2	47.7 (5)
P4—C61—C62—C63	177.4 (5)	C56—C55—P4—C61	152.2 (4)
C61—C62—C63—C64	1.8 (10)	C60—C55—P4—C61	−26.7 (5)
C62—C63—C64—C65	−0.7 (10)	C56—C55—P4—C67	−99.9 (5)
C63—C64—C65—C66	−0.5 (10)	C60—C55—P4—C67	81.2 (5)
C64—C65—C66—C61	0.7 (9)	C56—C55—P4—Ni2	32.1 (5)
C62—C61—C66—C65	0.4 (9)	C60—C55—P4—Ni2	−146.8 (4)

### Hydrogen-bond geometry (Å, º)

Cg2, Cg4, Cg6 and Cg8 are the centroids of the C7–C12, C19–C24, C31–C36 and C43–C8 rings, respectively.

**Table:**

*D*—H···*A*	*D*—H	H···*A*	*D*···*A*	*D*—H···*A*
C2—H2···Br1	0.95	2.90	3.779 (7)	155
C9—H9···Br1^i^	0.95	2.85	3.588 (6)	135
C38—H38···Br2	0.95	2.88	3.777 (7)	157
C45—H45···Br2^ii^	0.95	2.89	3.622 (6)	135
C72—H72···Br2	0.95	2.85	3.706 (6)	150
C18—H18···*Cg*4	0.95	2.88	3.681 (7)	143
C30—H30···*Cg*2	0.95	2.65	3.487 (7)	147
C35—H35···*Cg*4^iii^	0.95	2.90	3.711 (7)	145
C56—H56···*Cg*8	0.95	2.67	3.523 (7)	149
C64—H64···*Cg*6^iv^	0.95	2.69	3.490 (7)	142

Symmetry codes: (i) *x*+1, *y*, *z*; (ii) *x*−1, *y*, *z*; (iii) −*x*+1, −*y*+1, −*z*; (iv) −*x*+2, −*y*+1, −*z*.
