# Supplementary figures and images for: Crystal structure of bromido­nitro­syl­bis(tri­phenyl­phosphane-κP)nickel(II)
Source: Acta Crystallogr E Crystallogr Commun. 2015 Mar 18;71(Pt 4):m87–8. doi: 10.1107/S2056989015004703 (PMC4438846; doi:10.1107/S2056989015004703)

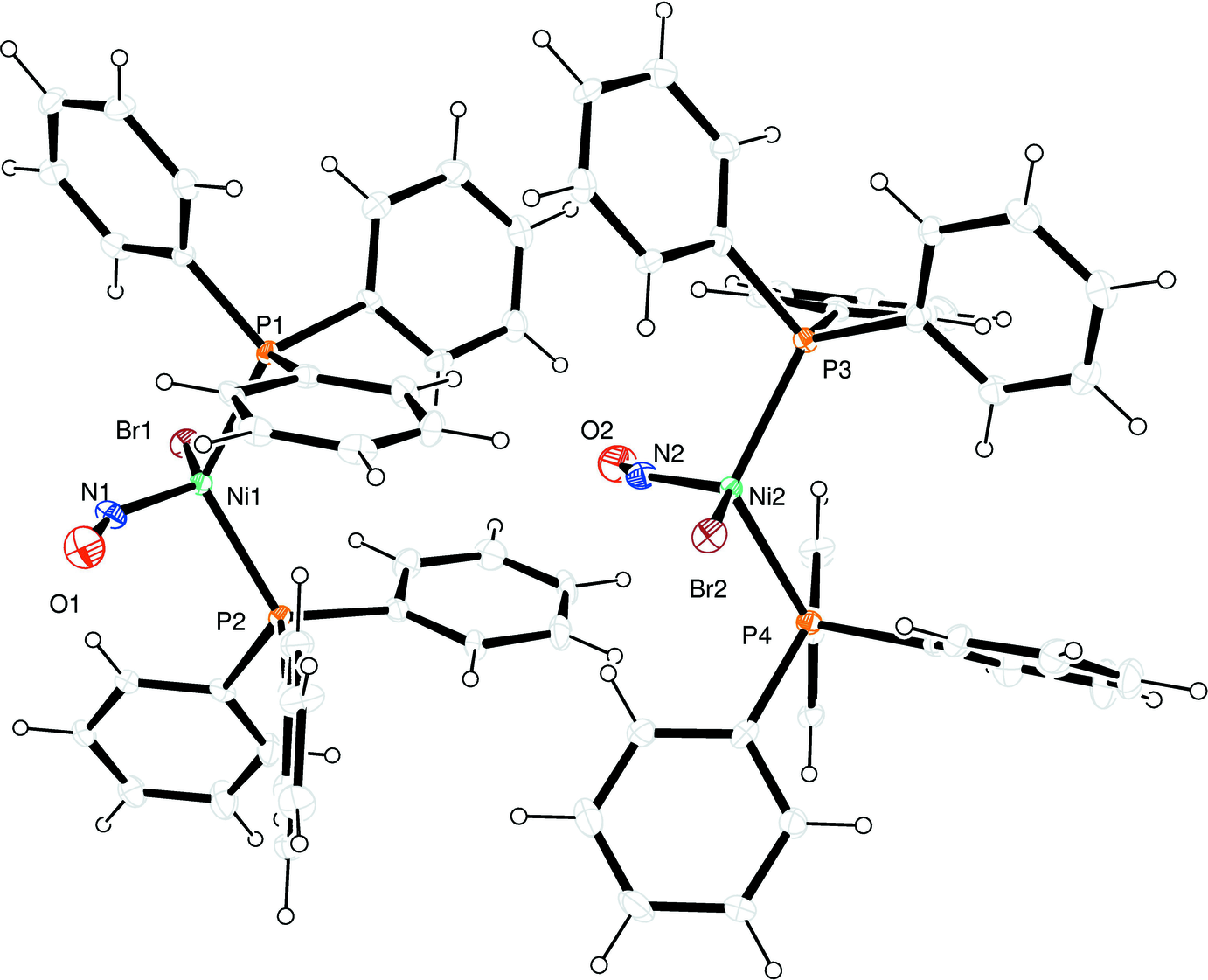

Supplement: Supplementary file 5 [file e-71-00m87-fig1.tif]

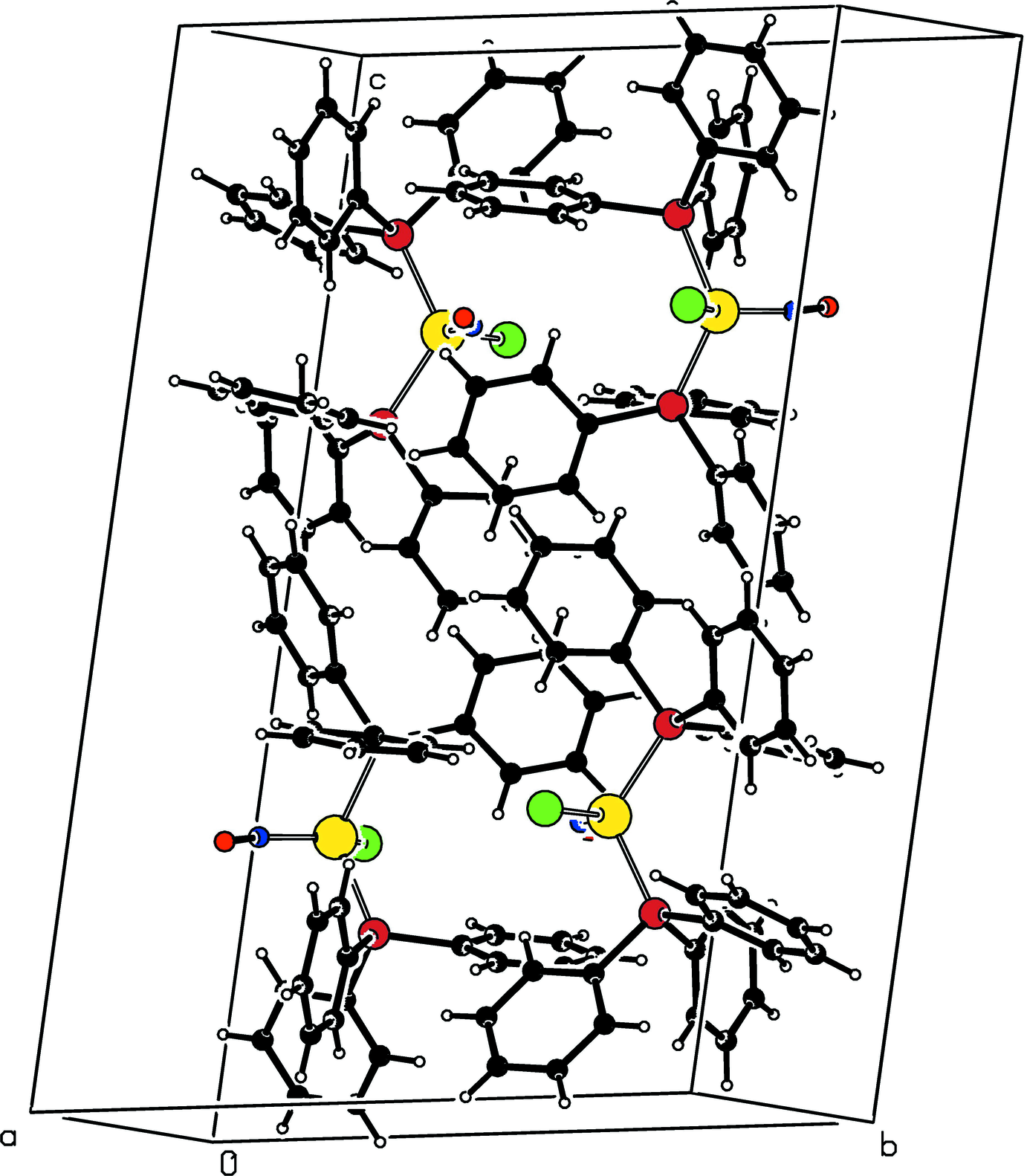

Supplement: Supplementary file 6 [file e-71-00m87-fig2.tif]
